# News Items Concerning Third Pan-American Medical Congress

**Published:** 1901-01

**Authors:** 


### NEWS ITEMS CONCERNING THIRD PAN-AMERICAN MEDICAL CONGRESS.

THE Organization Committee has postponed the meeting of the Third Pan-American Medical Congress until February 4th. It will be held from that date until the 7th, inclusive. All literature pertaining to the Congress is in Spanish, but occasional news items will be sent to the weekly journals.

Delegates can go either by the land routes, which are all via Florida, or by the water route from New York. Full particulars concerning these routes, excepting that of the Ward Line steamer from New York, will be found in the *A. M. A. Journal*, of November 3d. Those coming from south of Washington and west of Pittsburg, will probably select the Florida route, while those from the Northeast will find the Ward Line more convenient. The steamer Seguranza, of the Ward Line, leaves New York January 30th, arriving in Cuba on February 3d, the day before the beginning of the Congress. The new Ward Line steamer Morro Castle, holding one hundred and thirty-five cabin passengers, leaves Havana on February 9th, reaching New York February 11th. The round trip, everything included, is $60.00. Any one going via this route will be absent from New York twelve days.

Any of the delegates going down by the Ward Line who desire it, may, by a supplementary payment of $25.00, return via Santiago. This trip will take from ten to twelve days, during which time the passengers live on the ship, and have their meals there if they desire. The party goes from Havana by rail to Cienfuegos in one afternoon, there taking the steamer to Santiago, and from Santiago to Nassau in the Bahamas, and from Nassau to New York. The stay in these different ports will be for a day or more.

English-speaking members of the Cuban Reception Committee will meet all steamers on their arrival in Havana and escort the delegates to the hotels assigned to them. The best hotels in Havana are, Telegrafo, Mascotte, Inglaterra, and Pasaje. Rates, $3.00 to $5.00 a day.

The Entertainment Committee, appointed by the City Council of Havana, for the purpose of making it pleasant for the delegates during their stay, have arranged a program, which although as yet not fully determined upon in every detail, is nevertheless, one which adds an attractive feature to the meeting.

There will be a large ball given at the Tacon theater. This will be under the management of a sub-committee and an auxiliary ladies' committee. The committee is sparing no pains to make the ball the great social feature of the week, and one that will be most enjoyable, not only to the delegates, but also to any ladies in their parties. (There will be a banquet given at the same place.) On another day an excursion will be made to the sugar plantation of Mr. LaCosta, near Havana, on three government transports. On arriving there the delegates will be shown the manner of growing sugar-cane, gathering it, grinding, cooking, and refining the sugar derived therefrom; as well as all other points of interest connected with the method of making sugar, and life on a sugar estate. Refreshments will then be served either on the estate, or on the transports returning. These transports will be so arranged for the guests as to give the greatest amount of comfort and pleasure during the trip. On another day there will be a parade of the police and an exhibition of the fire companies, in which they will show the promptness and skill with which they combat any fires taking place in the city. Receptions will also be given by various officials, and private dinners by numerous Cuban physicians to the delegates with whom they are acquainted.

The three general sessions of the Congress, at which papers of general interest will be presented, and addresses delivered by official representatives from the different countries, will be held at the

Tacon theater, in Havana. The section meetings will be held in the University, on the first floor, and on the first and section floors of the Institute. The delegates from Mexico are at present in the lead, and a large number of abstracts have been received from them.

Mention will here be made of the names of the Presidents of the differents sections, all Cubans, as the Presidents are representatives of the country in which the Congress takes place, and the Secretaries for the United States. All communications and titles of papers should be sent to the United States Secretary of the Section covering the subject, or to the Associate Secretary for United States of the Congress, Dr. Ramon Guiteras, 75 W. 55th street, New York, N. Y. A preliminary program will be published shortly.

The officers of the different Sections are:

General Medicine—Dr. Carlos Finlay, president; secretary, Dr. Judson Daland, 317 S. 18th street, Philadelpha, Pa.

General Surgery—Dr. Tomas Plascencia, president; Dr. W. P. Nicolson, Atlanta, Ga., secretary.

Military Medicine and Surgery—Dr. Eugene Sancho Agramonte, president: Major Jefferson Kean, U. S. A., Quemados, Cuba, secretary.

Obstetrics—Dr. Eusebio Hernandez, president; Dr. Gustav Zinke, 13 Garfield Place, Cincinnati, Ohio, secretary.

Physiology—Dr. M. Sanchez Toledo, president; Dr. A. P. Brubaker, 105 W. 34th street, Philadelphia, Pa., secretary.

Pediatrics—Dr. Joaquin L. Duenas, president; Dr. I. N. Love, 49 W. 44th street, New York, N. Y., secretary.

Opthalmology—Dr. Enrique Lopez, president; Dr. John E. Weeks, 40 E. 57th street, New York, N. Y., secretary.

Gynecology and Abdominal Surgery—Dr. Gabriel Casusa, president; Dr. H. P. Newman, 103 State street, Chicago, Ill., secretary.

Therapeutics—Dr. Raimundo Castro, president; Dr. Hobart A. Hare, 222 S. 15th street, Philadelphia, Pa., secretary.

Pathology—Dr. Raimundo Castro, president; Dr. A. E. Thayer, 352 W. 117th street, New York, N. Y., secretary.

Anatomy—Dr. Federico Horsman, president; Dr. A. D. Bevan, 100 State street, Chicago, Ills., secretary.

Laryngology and Rhinology—Dr. Carlos Desvernine, president; Dr. G. H. Mekuen, 1419 Walnut street, Philadelphia, Pa., secretary.

Otology—Dr. Carlos Desvernine, president; Dr. J. F. McKernon, 62 W. 52d street, New York, N. Y., secretary.

Dermatology and Syphilography—Dr. Henry Robelin, president; Dr. A. Ravegli, 5 Garfield Place, Cincinnati, O., secretary.

General Hygiene and Demography—Dr. Vincents de la Guardia, president; Dr. Alva H. Doty, Quarantine Station, Staten Island, N. Y., secretary.

Marine Hygiene and Quarantine—Dr. Louis Cowley, president; Dr. H. M. Woodward, Surgeon M. H. S., Washington, D. C., secretary.

Orthopedic Surgery—Dr. Tomas Plascencia, president; Dr. John Ridlon, 103 State street, Chicago, Ill., secretary.

Mental and Nervous Diseases—Dr. Gustavo Lopez, president; Dr. Chas. H. Hughes, 3857 Olive street, St. Louis, Mo., secretary.

Dental and Buccal Surgery—Dr. Erastus Wilson, president; Dr. Eugene Talbot, Columbus Memorial Building, Chicago, Ill., secretary.

Medical Jurisprudence—Dr. Jose M. Cespedes, president; Dr. H. A. West, Galveston, Texas, secretary.

Medical Pedagogy—Dr. Manuel Delfin, president; Dr. Otis K. Newell, 13 Central Park West, New York, N. Y., secretary.

Railway Surgery—Dr. Tomas Plascencia, president; Dr. Duncan Eve, 700 Church street, Nashville, Tenn., secretary.

